# M-AMST: an automatic 3D neuron tracing method based on mean shift and adapted minimum spanning tree

**DOI:** 10.1186/s12859-017-1597-9

**Published:** 2017-03-29

**Authors:** Zhijiang Wan, Yishan He, Ming Hao, Jian Yang, Ning Zhong

**Affiliations:** 1 Beijing Advanced Innovation Center for Future Internet Technology, Beijing University of Technology, Beijing, China; 2 Department of Life Science and Informatics, Maebashi Institute of Technology, Maebashi, Japan; 3 International WIC Institute, Beijing University of Technology, Beijing, China; 4 Beijing Key Laboratory of MRI and Brain Informatics, Beijing, China; 5 Beijing International Collaboration Base on Brain Informatics and Wisdom Services, Beijing, China

**Keywords:** M-AMST, Neuron reconstruction, Mean shift, Sphere model, Coordinate transformation

## Abstract

**Background:**

Understanding the working mechanism of the brain is one of the grandest challenges for modern science. Toward this end, the BigNeuron project was launched to gather a worldwide community to establish a big data resource and a set of the state-of-the-art of single neuron reconstruction algorithms. Many groups contributed their own algorithms for the project, including our mean shift and minimum spanning tree (M-MST). Although M-MST is intuitive and easy to implement, the MST just considers spatial information of single neuron and ignores the shape information, which might lead to less precise connections between some neuron segments. In this paper, we propose an improved algorithm, namely M-AMST, in which a rotating sphere model based on coordinate transformation is used to improve the weight calculation method in M-MST.

**Results:**

Two experiments are designed to illustrate the effect of adapted minimum spanning tree algorithm and the adoptability of M-AMST in reconstructing variety of neuron image datasets respectively. In the experiment 1, taking the reconstruction of APP2 as reference, we produce the four difference scores (entire structure average (ESA), different structure average (DSA), percentage of different structure (PDS) and max distance of neurons’ nodes (MDNN)) by comparing the neuron reconstruction of the APP2 and the other 5 competing algorithm. The result shows that M-AMST gets lower difference scores than M-MST in ESA, PDS and MDNN. Meanwhile, M-AMST is better than N-MST in ESA and MDNN. It indicates that utilizing the adapted minimum spanning tree algorithm which took the shape information of neuron into account can achieve better neuron reconstructions. In the experiment 2, 7 neuron image datasets are reconstructed and the four difference scores are calculated by comparing the gold standard reconstruction and the reconstructions produced by 6 competing algorithms. Comparing the four difference scores of M-AMST and the other 5 algorithm, we can conclude that M-AMST is able to achieve the best difference score in 3 datasets and get the second-best difference score in the other 2 datasets.

**Conclusions:**

We develop a pathway extraction method using a rotating sphere model based on coordinate transformation to improve the weight calculation approach in MST. The experimental results show that M-AMST utilizes the adapted minimum spanning tree algorithm which takes the shape information of neuron into account can achieve better neuron reconstructions. Moreover, M-AMST is able to get good neuron reconstruction in variety of image datasets.

## Background

Understanding how the brain works from the aspect of cognition and structure is one of the greatest challenges for modern science [1]. On one hand, it is meaningful to systematically investigate human information processing mechanisms from both macro and micro points of views by cooperatively using experimental, computational, cognitive neuroscience. On the other hand, acquiring knowledge of the neuron’ morphological structure is also of particular importance to simulate the electrophysiological behavior which intricately links with cognitive function and promotes our understanding of brain. Based on the above views, several research journals (Brain Informatics [2], Bioimage Informatics [3] and Neuroinformatics [4]), worldwide neuron reconstruction contest (DIADEM [5]) and bench testing project (BigNeuron [6]) have been launched. One of their basic tasks is how to extract the neuronal morphology from the molecular and cellular microscopic images, namely neuron reconstruction or neuron tracing.

In order to get the neuron tracing algorithms with high performance as many as possible, the BigNeuron project aims at gathering a worldwide community to define and advance the state-of-the-art of single neuron reconstruction. The primary method to achieve that goal is to bench test as many varieties of automated neuron reconstruction methods as possible against as many neuron datasets as possible following standardized data protocols [6]. So far, varieties of neuron reconstruction methods based on image segmentation theories such as fuzzy set [7], level set [8, 9], active contour model [10–12], graph theory [13], and clustering [14, 15] have been contributed to the project. For example, APP2 algorithm based on level set theory can generate reliable tree morphology of neuron with the fastest tracing speed [9]. A neuron tracing algorithm named Micro-Optical Sectioning Tomography ray-shooting (MOST for short) achieves a good result in terabytes 3D datasets of the whole mouse brain [16]. Additionally, a neuron tracing algorithm named SIMPLE is a DT-based method and can produce better reconstruction in dragonfly thoracic ganglia neuron images than other methods [17]. A neuron tracing method based on graph theory, namely neuron tracing minimum spanning tree (N-MST for short), also gets reasonable reconstructions for several neuron image datasets. Due to the spatial nature of image, the methods mentioned above are all take the spatial information into account. However, in some segmentation scenarios, the objects of interest may be reasonably characterized by an intensity distribution. In the 3D image, the more voxels distributed in an image region, the region has a higher voxel density. For such a situation, it is important to integrate intensity information into a spatial algorithm. The neuron tracing method based on clustering is the algorithm which adopts spatial information and intensity distribution of neuron simultaneously. Moreover, because the clustering algorithms are intuitive and, some of them, easy to implement, they are very popular and widely used in image segmentation. For instance, mean shift is a nonparametric density gradient estimation using a generalized kernel approach and is one of the most powerful clustering techniques. Cai et al. proposed a cross-sections of axons detection and connection method using nonlinear diffusion and mean shift [14]. The automatic method can shift the centroids of cross-section on slice A iteratively until the sample mean convergence on slice B. They concluded the centroid on slice A and the centroid on slice B correspond to the same axon. Comaniciu et al. proposed a robust approach for the analysis of a complex multimodal feature space and to delineate arbitrarily shaped clusters in it [18]. The basic computational module of the technique is the mean shift. They proved for discrete data the convergence of a recursive mean shift procedure to the nearest stationary point of the underlying density function and, thus, its utility in detecting the modes of the density. They also claimed that the mean shift algorithm is a density estimation-based non-parametric clustering approach that the data space can be regarded as the empirical probability density function (p.d.f) of the represented parameter. As we know, dense region in the data space corresponds to local maxima of the p.d.f, that is, to the modes of the unknown density. Once the location of a mode is determined, the cluster associated with it is delineated based on the local structure of the data space. As it happens, the neuron image generated by fluorescent probes has the characteristics of spatial distribution, intensity discretization and the portions around the neuron skeleton have a higher voxel density. Based on this, we developed a neuron tracing algorithm based on mean shift and minimum spanning tree as a contribution to the BigNeuron project in the beginning. Specifically, the algorithm can move each voxel to the local mean until automatically get the convergence region which has the local maxima of the p.d.f. Meanwhile, the voxels in the convergence regions can also be considered as the classification voxels which indicate the modes of unknown density, other voxels which shift toward and finally locate at the regions after several iterations can be marked as the same classification as the corresponding classification voxel. They also can be regarded as the voxels subordinated to the classification voxels. Based on the subordinate voxels belong to the different classification voxel, the local structure of the neuron can be delineated. In this method, not only the information of voxel density distribution of neuron image can be captured correctly, but also got the sufficient voxels belong to several modes to delineate the whole neuron topological structure. In the basis of the classification voxels and their own subordinate voxels, we can use the minimum spanning tree (MST) algorithm to reconstruct the neuron. It is worth noting that with MST, the information of spatial distribution of neuron is also adopted to get the neuron reconstruction.

However, the experimental result of M-MST shows that although the M-MST algorithm can reconstruct 120 images successfully, it generates less precise connection between some neuron segments since the MST just takes the spatial distance as the weight of edge to build the paternity between nodes. The node is defined as the voxel with the property of spatial coordinates, radius, node type and parent compartment. The situation about less precise connection between some neuron segments caused by MST can be illustrated as the following:

Node A and node B belong to the different neuron segments and have the nearest distance or the smallest edge weight in the neuron image. According to the topological structure of original neuron image, there is a gap between them. In this case, it is not suitable to use the minimum spanning tree algorithm to build paternity of the two nodes directly. If we can detect the gap between the two nodes and set their weight of edge in MST as a high value which is exceed a lot than the real spatial distance, the MST will choose other pair of nodes which have no gap between them to form the neuron segment. The pair of nodes which have no gap between them are more likely subordinate to the same neuron segment. Once the gap is detected, the shape information can be captured. Therefore, the weight calculation method considers the shape information of neuron will help for achieving more precise neuron reconstruction.

**Fig. 1:**
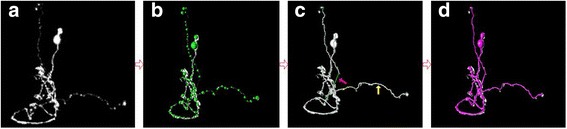
Overview of the M-AMST and the related reconstruction result in four steps. **a** an original neuron image. **b** the node set in green color extracted by mean shift algorithm and pruned by the node pruning method are overlaid on top of original neuron image. **c** the initial reconstruction result is overlaid on top of original neuron image, the white line between green nodes pointed by the yellow arrow is the pathway extracted by the rotating sphere model based on coordinate transformation, the green line is not a pathway generated by the model since there is a gap between the two nodes. **d** final reconstruction result in red color is overlaid on top of original neuron image

In this paper, we focus on introducing an improved algorithm, namely M-AMST, in which a rotating sphere model based on coordinate transformation is used to improve the weight calculation method in M-MST. Figure 1 gives an overview of the M-AMST and the related reconstruction result in four steps. The four steps can be described as follows. Firstly, input a single neuron image (Fig. 1(a)) into the Vaa3D [19]. Secondly, for each foreground voxel, the mean shift algorithm defines a window with certain spatial range and takes the voxel as the center of the window. Then it shifts the voxel to the local mean iteratively until getting the convergence region. It is worth noting that the voxel located at the convergence region cannot shift for one step. This kind of voxel could be considered as the classification voxel. By observing their position in the neuron image, the most of classification voxels are located around the neuron skeleton, in which the neuron segment with high intensity and voxel density located. Moreover, a radius calculation method is adopted to calculate the radius of every foreground voxel. The foreground voxel with radius can be called as foreground node. Due to the size of every foreground node is greater or equal than 1.0, some of them might be overlapped or even covered by others. Such case is deemed as the repeat expression or the over reconstruction of neuron. We need the nodes as few as possible to express the neuronal morphology as complete as possible. In response, a node pruning method based on the distance between the pair of nodes and their own radiuses is developed. After that, a slew of nodes can be retained and formed to be a node set. The node set will be considered as the seeds to be input into the adapted MST to build the tree structure of neuron. In Fig. 1(b), the node set in green color extracted by mean shift algorithm and pruned by the node pruning method are overlaid on top of original neuron image. Thirdly, a rotating sphere model based on coordinate transformation is implemented to extract a pathway between each pair of nodes. In Fig. 1(c), the initial reconstruction result is overlaid on top of original neuron image, the white line between green nodes pointed by the yellow arrow is the pathway extracted by the rotating sphere model based on coordinate transformation, the green line is not a pathway generated by the model since there is a gap between the two nodes. Fourthly, take the accumulating distance of the node list in the pathway between each pair of nodes as the weight and use the minimum spanning tree algorithm to reconstruct the neuron image. In Fig. 1(d), final reconstruction result in red color is overlaid on top of original neuron image.

## Method

### Topological structure segmentation

#### A. Voxel clustering using mean shift algorithm

On the whole, we follow the sequence of neuron reconstruction operations: binarization, skeletonization, rectification and graph representation [1]. In the binarization operation, we firstly define the voxel whose intensity is less than a threshold as the dark spot and otherwise the bright spot. For each voxel, the number of the dark spots and the bright spots among the 26 surrounding voxels are calculated respectively. And then, we calculate the ratio of the number of the dark spots and the bright spots, the ratio is compared with a threshold to determine whether the voxel is a foreground or not. In the skeletonization operation, we use mean shift algorithm to extract the neuron skeleton.

The implementation of mean shift in this paper is interpreted as the following steps:

1. Mean shift involves shifting a kernel iteratively to a region with higher density until convergence. We shift the 3D coordinate of each voxel using a Gaussian kernel described as the following:

1 $$ \mathrm{K}\left(\mathrm{x}\right)=\frac{1}{2\pi {\delta}^2} \exp \left(- C*\frac{{\left| x-\overline{x}\right|}^2}{2{\delta}^2}\right), $$

C is a scaling coefficient, $\overline{x}$ is the average and *δ* is standard deviation. The calculation method of $\overline{x}$ and *δ* are illustrated as follow.

(2) Assume a sphere centered on voxel P and with radius r. Using X-axis as example, the x in the formula (1) can be calculated by

2 $$ \mathrm{x}=\left({x}^r-{x}_i^r\right)*\left({x}^r-{x}_i^r\right)/ r* r, $$

where *x* ^*r*^ means the x-coordinate of P, *x*_*i*_^*r*^ means the x-coordinate of a voxel in the sphere. The standard deviation *δ* is calculated by

3 $$ \updelta =\sqrt{{\displaystyle {\sum}_{i=1}^n}\left({x}_i-\overline{x}\right)*\left({x}_i-\overline{x}\right)/ n}, $$

where *x*_*i*_ is a x-coordinate converting value which is obtained by formula (2), $\overline{x}$ is the average of the x-coordinate converting value of every voxel in the sphere. The average $\overline{x}$ is calculated by

4 $$ \overline{x}={\displaystyle {\sum}_{i=1}^n}{x}_i/ n. $$

(3) The new coordinate value of the sphere center in X-axis is calculated by

5 $$ {\mathrm{next}}_x=\frac{{\displaystyle {\sum}_a} K\left(\left({x}^r-{x}_i^r\right)*\left({x}^r-{x}_i^r\right)/ r* r\right)*{x}_i^r}{{\displaystyle {\sum}_a} K\left(\left({x}^r-{x}_i^r\right)*\left({x}^r-{x}_i^r\right)/ r* r\right)}. $$

where next_x_ is the coordinate values of the new center voxel in X-axis. The symbol a indicates the whole sphere region for the current foreground voxel. It is worth noting that the calculation method of the new coordinate value in Y-axis and Z-axis is as the same as the method mentioned above.

#### B. Covered node pruning

As mentioned above, we define the voxels which cannot shift for one iteration as the classification voxels which also can be called as marks, the other voxels that shift toward iteratively and finally located at the marks can be defined as the corresponding subordinate voxels. The two kinds of voxels can be used to reconstruct the whole neuronal topological structure. However, after calculating radius for the marks and the corresponding subordinate voxels, they might be overlapped or even covered by others. In order to get the nodes as few as possible to express as complete neuronal morphology as possible, we prune the marks and other nodes overlapped or covered by others using a node pruning method. The node pruning method adopts three steps listed as follows:

1. For a pair of marks, prune the covered mark according to the distance between them and their own radiuses. Figure 2 gives three covering situations of mark. The red and purple dots represent two different marks and their own radiuses are r_1_ and r_2_ respectively. We define two marks should all be kept (Fig. 2a) if the difference between their Euclidean distance D and the sum of their radiuses is greater than a threshold. Conversely, prune one of marks (Fig. 2b and c) without defining a particular pruning priority. The threshold should be set greater than 3 according to the prior knowledge which claimed that the human eye can tell the detail variation above 3 voxels.

**Fig. 2:**
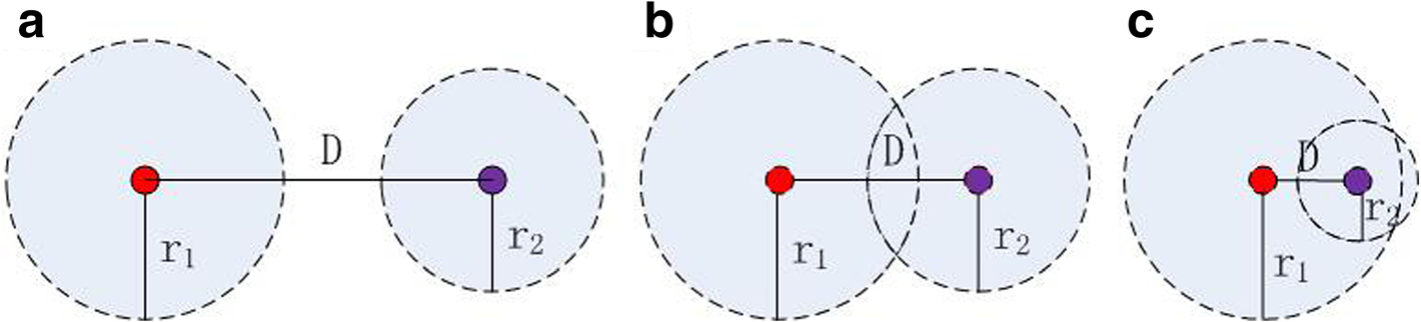
Three covering situations of mark. Red and purple dots represent two different marks, their own radiuses are r_1_ and r_2_ respectively. D is their Euclidean distance. **a** Keep; **b** prune one mark and **c** prune one mark
2. Remark the subordinate nodes of the removing mark to the corresponding keeping mark. Specifically, due to some covered or overlapped marks are pruned, the nodes which are subordinate to the removing marks should be re-subordinate to the keeping marks. We deal with this using a two-fold QMap data structure, the keys of first and second QMap mean the order number of keeping mark and the removing mark in the original mark set respectively. The values of second QMap mean the subordinate node set belongs to the corresponding removing mark. After remarking, we get the keeping marks and their own subordinate node set.
3. For each node set, prune the subordinate nodes overlapped or covered by others but always keep the marks. The pruning method is consistent with the first step.

### Pathway extraction using a sphere model

Based on the marks and their own subordinate node set, we use MST algorithm to reconstruct the neuron and finish the graph representation step. The main principle of MST is to select a pathway with minimum total weights to connect all vertices in a connected and undirected graph. Taking the spatial distance between each pair of nodes as the weight is the most obvious weight calculation method. In M-MST, we build an undirected graph by connecting all extracted nodes firstly and then calculate the spatial distance between each pair of nodes as the weight of edge. However, building the paternity between each pair of nodes according to their spatial distance could easily lead to the less precise connection between some neuron segments. The weight calculation method combines the shape and spatial information of neuron will help for achieving more precise neuron reconstruction. Therefore, in order to get an accurate neuronal topological reconstruction, a rotating sphere model based on coordinate transformation is proposed to improve the weight calculation method of edge in M-MST. The core idea of the rotating sphere model is to move the sphere centered on each node in the node set to progressively approach other nodes. For each pair of nodes, we define the two nodes as starting node and terminal node respectively. A pathway could be extracted between the starting node and the terminal node if there is no gap between them or one node is not far away from the other. The main principle of the rotating sphere model based on coordinate transformation is described as the following.

For each pair of nodes, a sphere centered at the starting node can be constructed in the beginning. The sphere can be split up into several quadrants by the 3D coordinate axis, every quadrant contains several foreground voxels. We always define the line between the starting node and terminal node as the Y’-axis and the positive direction of Y’-axis starts from the starting node to the terminal node. Taking the position of starting node as reference, different foreground voxels in the sphere have different coordinate value of Y’-axis. Suppose that there are two vectors named A and B respectively. The vector A starts from the sphere center to the foreground voxel with positive coordinate value of Y’-axis, the vector B starts from the sphere center to the voxel with negative coordinate value of Y’-axis. The angle between vector A and the positive direction of Y’-axis will always less or equal than 90° and the cosine value of the angle is greater or equal than 0. The angle between vector B and the positive direction of Y’-axis will always greater than 90° and the cosine value of the angle is less than 0. We define the rotating direction of the sphere always follow the direction of the vector A. Due to the Vaa3D platform who possesses a self-defined 3D coordinate system (X, Y and Z), the coordinate value of every foreground voxel in the 3D coordinate system should be transformed to the new coordinate system (X’, Y’ and Z’). This operation aims at ensuring the sphere centered at the starting node could always be approaching to the terminal node. Toward this purpose, we use two rotation steps to re-calculate the new coordinate value of every foreground voxel in the sphere.

1. First rotation:

6 $$ \begin{array}{c}\hfill {x}_1= x\hfill \\ {}\hfill {y}_1= cos\theta * y- sin\theta * z\hfill \\ {}\hfill {z}_1= sin\theta * y+ cos\theta * z\hfill \end{array} $$

where *θ* means the angle between Y-axis and T1, T1 means the line between the projection point of terminal node in the YOZ plane and sphere center.

(2) Second rotation:

7 $$ \begin{array}{c}\hfill {x}_2= cos\gamma *{x}_1- sin\gamma *{y}_1\hfill \\ {}\hfill {y}_2= sin\gamma *{x}_1+ cos\gamma *{y}_1\hfill \\ {}\hfill {z}_2={z}_1\hfill \end{array} $$

where *γ* means the angle between Y’-axis and T2, T2 means the line between the projection point of terminal node in the YOZ plane and sphere center.

It is worth noting that in every rotating step, we guarantee the new sphere center located at the voxel which intensity is greater than 0. Specifically, we select a foreground voxel which intensity is greater than 0 from one of the quadrants as the new center of sphere. Every foreground voxel in different quadrant can be assigned a weight which indicates the gravitation attracted by the terminal node. We accumulate the weight value of all voxels in every quadrant respectively and select the maximum as the candidate quadrant, the new center of sphere is the voxel which has the shortest distance to the terminal node in the candidate quadrant. Every step will iteratively repeat the method until the terminal node located within the radius range of the new sphere. The calculation formula of total gravitation F for each quadrant is shown as the following:

8 $$ \mathrm{F}={\displaystyle {\sum}_q} c o s\theta \ast \left({I}_1\ast {I}_2/{D}^2\right)/ n $$

where *θ* means the angle between the vector which is from the sphere center to the foreground voxel and the positive direction of Y’-axis. I_1_ and I_2_ indicate the intensity of the foreground voxel in the quadrant and terminal node respectively. D is the Euclidean distance between the foreground voxel and terminal node, n is the number of voxels included in the q-th quadrant.

**Fig. 3:**
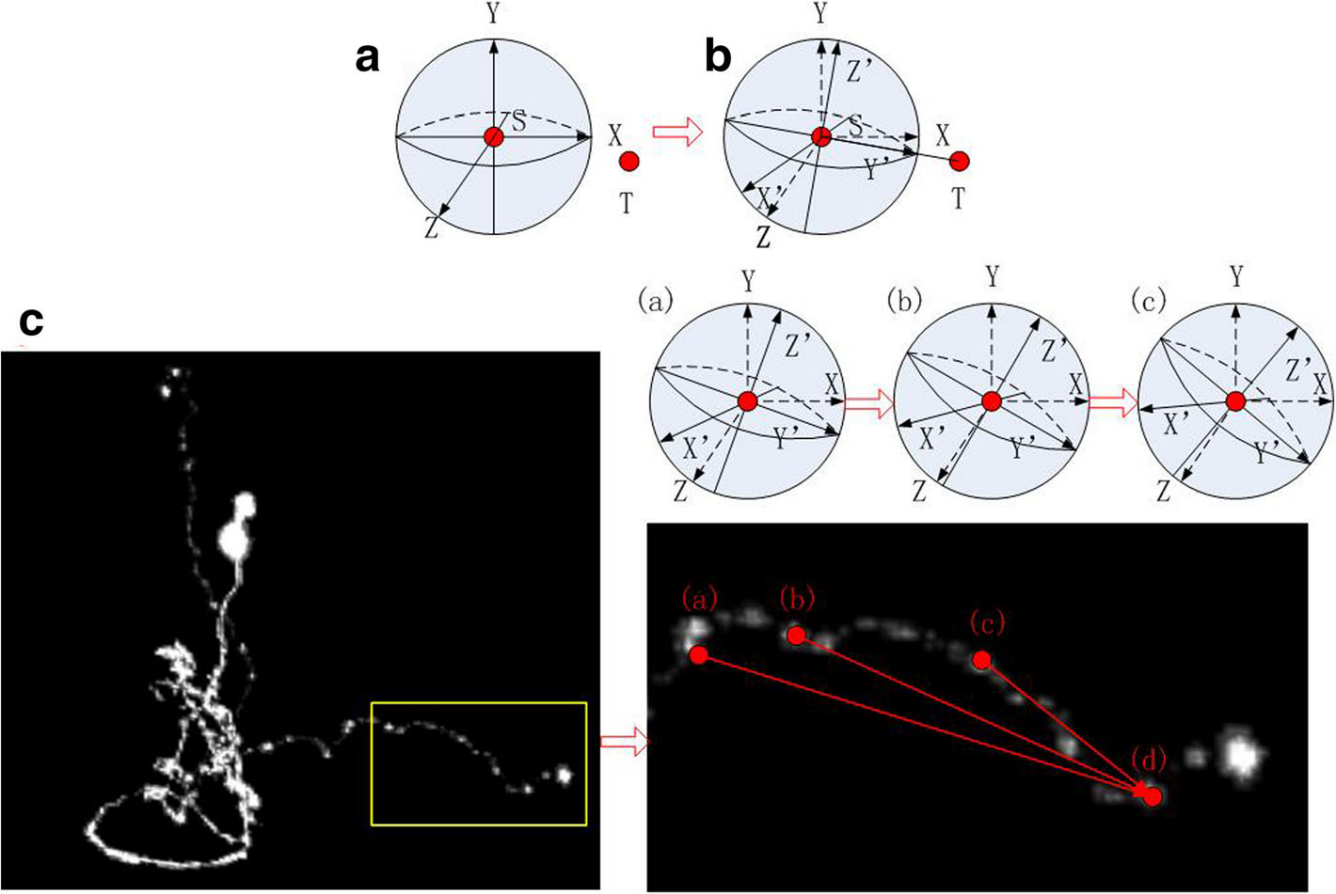
The schematic map of the rotating sphere model. Part A indicates the coordinate system (X, Y and Z) in Vaa3D platform, the two red dots (S and T) imply the starting and terminal node respectively. Part B means the new coordinate system (X’, Y’ and Z’). Part C illustrates the rotating procedure from the starting voxel (marked with red (**a**)) to the terminal voxel (marked with red (**d**)). Red (**b**) and red (**c**) are the schematic sphere center which are selected from the rotating procedures

Figure 3 gives the schematic map of the rotating sphere model. Part A indicates the coordinate system (X, Y and Z) in Vaa3D platform, the two red dots (S and T) imply the starting and terminal node respectively. Part B means the new coordinate system (X’, Y’ and Z’). Part C illustrates the rotating steps from the starting node (marked with red (a)) to the terminal node (marked with red (d)). Red (b) and red (c) are the schematic sphere center which are selected from the rotating procedures. Using the rotating sphere model based on coordinate transformation, a pathway can be extracted if there is no gap between the pair of nodes. It is worth noting that the pathway contains a node list since the sphere model continuously selects the foreground voxel as the new sphere center in every rotating step. Every node list represents the shape information between the corresponding pair of nodes.

### Neuron reconstruction adopting MST algorithm

For each rotating step, we take the sphere center chose in the previous rotating step as the parent node of the current sphere center. A node list can be got if the starting node approaches the terminal node successfully. The node list is also a list which is composed of voxels with paternity. We defined the node list as the pathway between the starting node and the terminal node. Comparing with using the Euclidean distance as the weight of edge of the MST, it is more reasonable to adopt the accumulating distance of the node list as the weight of edge. Specifically, the accumulating distance can be calculated by summing up the distance between each pair of the parent node and the child node in the list. And then, take the accumulating Euclidean distance as the weight of edge of the MST. Notable, the weight will be set to a numerical value which is far greater than the real spatial distance if the sphere rotating model fails to extract the pathway. The reason for the sphere rotating model failed can be concluded as two aspects, the one is a gap exists between the two nodes, the other is the rotating times beyond the pre-configured threshold since one node is far away from the other. The calculation method of the weight in M-AMST descripted as the following:

if pathway exist,

then *W*_*E*_ = ∑_*i* = 1_^*n* − 1^ *Dis*(*p*_*i*_, *p*_(*i* + 1)_);

else *W*_*E*_ = *M* ∗ Dis(S, T),

where W_E_ is the accumulating Euclidean distance of the pathway between the corresponding pair of nodes, p_i_ and p_i+1_ mean the child node and parent node in the node list respectively, Dis indicates the Euclidean distance between the two nodes, M means a positive integer value which is greater than 10.

After that, we use MST algorithm to build a graph representation in SWC format. Specifically, the weights between all pairs of nodes can form a weight diagonal matrix, which can be acted as the input of MST algorithm. In order to reconstruct the neuron image more elaborate, based on the neuron reconstruction by MST, we fulfill the node list into the pathway between the corresponding pair of nodes.

## Results

### Parameters in the implementation

We implemented the M-AMST algorithm as a plugin of the Vaa3D which is the common platform to implement algorithms for BigNeuron project (bigneuron.org) bench testing. On the whole, the implementation of the M-AMST algorithm can be split into four steps which are summarized as follows:

1. Binarization. We define the voxel whose intensity which is less than a threshold as the dark spot and otherwise the bright spot. For each voxel, the number of the dark spots and the bright spots among the 26 surrounding voxels are calculated respectively. And then, we calculate the ratio of the number of the dark spots and the bright spots, the ratio is compared with a threshold to determine whether the voxel is a foreground or not. The intensity threshold and ratio threshold are set to be 30 and 0.3 respectively.
2. Skeletonization. For each foreground voxel, the mean shift algorithm defines a sphere with certain spatial range and takes the voxel as the center of the sphere. Then it shifts the voxel to the local mean iteratively until getting the convergence region. In order to avoid the endless loop, the number of shifting times of the foreground voxel is set to 100. The radius of the sphere is set to 5.
3. Rectification. We prune the nodes yielded from skeletonization step using a node pruning method. For a pair of nodes, we define two nodes should all be kept if the difference between their Euclidean distance D and the sum of their radiuses is greater than a certain voxel distance. The voxel distance is set to 3.
4. Graph representation. In the implementation of the rotating sphere model, the radius of sphere is set to 2 and the sphere was split into 4 quadrants. We extract the pathway between each pair of nodes and calculate the length of it by accumulating the Euclidean distance between the parent node and child node in the node list. A weight diagonal matrix can be generated to act as the input of MST algorithm. Based on the neuron reconstruction by MST, we fulfill the node list into the pathway between the corresponding pair of nodes. After that, in order to remove the unnecessary or redundant spurs in the tree structure, we adopt a hierarchical pruning method utilized in APP2. The code can be downloaded from www.github.com/Vaa3D/vaa3d_tools/tree/master/bigneuron_ported/APP2_ported.

### Experimental results

#### A. Neuron reconstruction efficiency comparison between M-AMST and M-MST

**Fig. 4:**
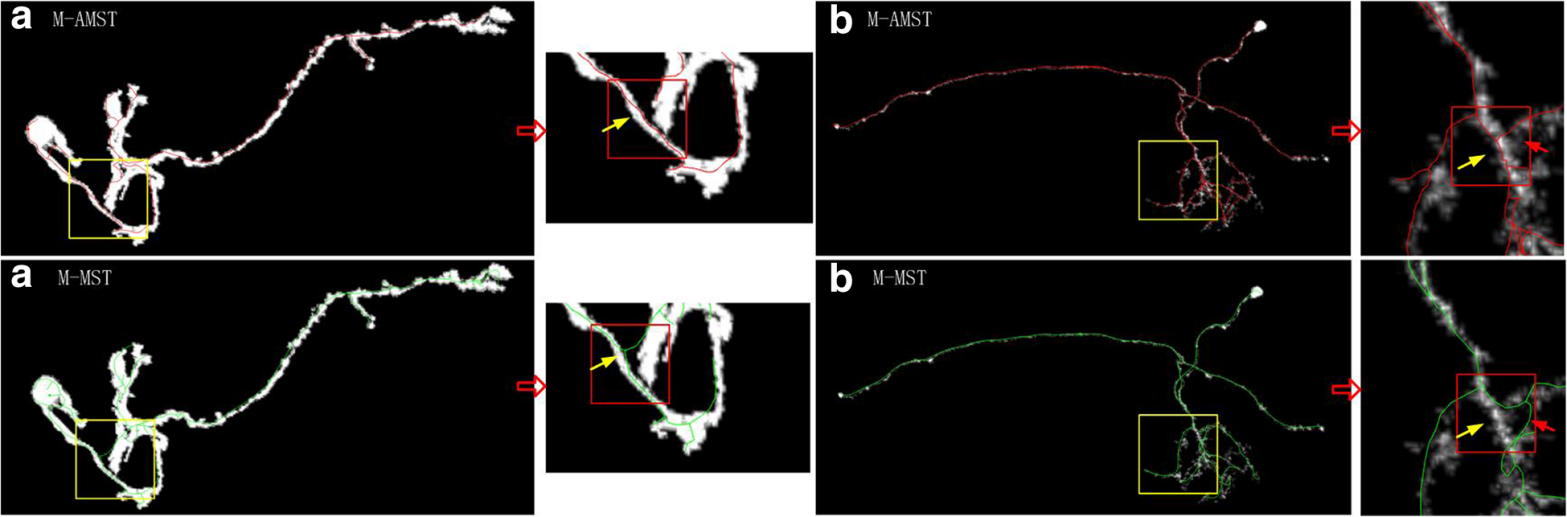
The comparison of neuron reconstructions using M-AMST and M-MST for two Drosophila neuron images. The reconstructions are overlaid on top of the original images for better visualization. The yellow box points out the different reconstruction results and the red box with the arrow make them more clearly

One hundred twenty confocal neuron images of the Drosophila were used to test the performance of M-AMST. Firstly, we selected two Drosophila neuron images from 120 images randomly to illustrate the difference between the reconstruction of the M-AMST and M-MST. The reconstruction results showed in Fig. 4 are overlaid on top of the original images for better visualization. For the first neuron image showed in the left part of Fig. 4, the yellow box contains the reconstruction results of the two methods and we can see them more clearly in the red box. For the result of M-MST displayed in the bottom left, the reconstruction is not accurate since it crosses the gap and connects two different segments (see green line and yellow arrow), but the result of the M-AMST shows in the up left is accurate. For the second neuron image showed in the right part of Fig. 4, there are two different parts which are showed in the red box with yellow and red arrow respectively. For the M-MST, a neuron portion is not reconstructed (see yellow arrow) successfully and the different neuron segments are connected by crossing the gap (see red arrow). The M-AMST method does not generate such bug and reconstructs the neuron image accurately. The result showed above proved that the reconstruction effect of M-MST is worse than M-AMST since the M-MST just considers the spatial distance between neuron segments and ignores the shape information of neuron morphology.

#### B. Running time comparison between M-AMST and M-MST

It is notable that all of the experiments were performed on a Panasonic laptop with 2.6 GHz Intel Core i5-4310U CPU and 4G RAM. Table 1 summarizes the running speed of M-AMST versus M-MST for several Drosophila neuron images. The running time of each step (binarization, skeletonization, rectification and graph representation) is represented by Tb, Ts, Tr and Tg respectively. M-AMST and M-MST indicates their own running time. Since the whole procedure of M-AMST is coherent with M-MST except the step of graph representation, we can envisage that the computational time of the adapted MST exceeds the original MST. The relative high computational complexity of M-AMST could be attributable to the efficiency of pathway extraction based on the sphere model. However, for some images, the running time of M-MST is measly outperformed M-AMST, which can be explained that the running time is affected by the dynamic change of laptop performance. Comparing with Tb, the running time of pathway extraction based on sphere model is not the arch time consuming step.

**Table 1:** Comparison of running time (seconds) of M-AMST and M-MST on few images

ID	Size	Memory (KB)	Running time (s)					
			Tb	Ts	Tr	Tg	M-AMST	M-MST
1	1024*1024*119	121857	35.147	0.046	0.016	4.384	39.593	38.438
2	1024*1024*109	111617	30.748	0.124	0.094	3.292	34.258	35.428
3	1024*1024*121	123905	33.494	0.156	0.078	5.647	39.375	33.431
4	1024*1024*111	113665	30.14	0.078	0.015	5.523	35.756	32.948
5	1024*1024*109	111617	31.138	0.031	0.031	5.835	37.035	33.119
6	1024*1024*125	128001	39.344	0.062	0.078	1.217	40.701	36.629
7	1024*1024*128	131073	36.115	0.062	0.031	3.838	40.046	40.155
8	1024*1024*115	117761	29.859	0.015	0.016	1.451	31.341	33.041
9	1024*1024*112	114689	31.527	0.032	0.031	3.245	34.835	30.577
10	1024*1024*117	119809	31.84	0.031	0.016	3.104	34.991	32.542

#### C. Comparison with other reconstruction algorithms

We compared the 120 neuron reconstructions generated by M-AMST with another four tracing algorithms which listed as follows: M-MST, MOST, SIMPLE, N-MST. Notably, the four tracing algorithms are also developed by the member groups of BigNeuron and the corresponding code can be downloaded from www.github.com/Vaa3D/vaa3d_tools/tree/master/bigneuron_ported. Due to the APP2 tracing algorithm is the fastest tracing algorithm and is reliable in generating tree morphology of neuron among the existing methods, we select the reconstructions generated by APP2 as the reference to compare the effect of the five algorithms. Moreover, we calculate four difference scores (ESA, DSA, PDS and MDNN) of the reconstructions produced by APP2 and the five tracing algorithms. Correspondingly, the four difference scores measure the overall average spatial divergence between two reconstructions, the spatial distance between different structures in two reconstruction, and the percentage of the neuron structure that noticeably varies in independent reconstruction, as well as the maximum distance to the nearest reconstruction elements between two reconstructions. The smaller value the four difference scores get, the neuron reconstruction effect of the competing algorithm is closer to the reference algorithm. To make a fair comparison, the reported results of the competing algorithms correspond to the default parameters called by respective plugins.

**Fig. 5:**
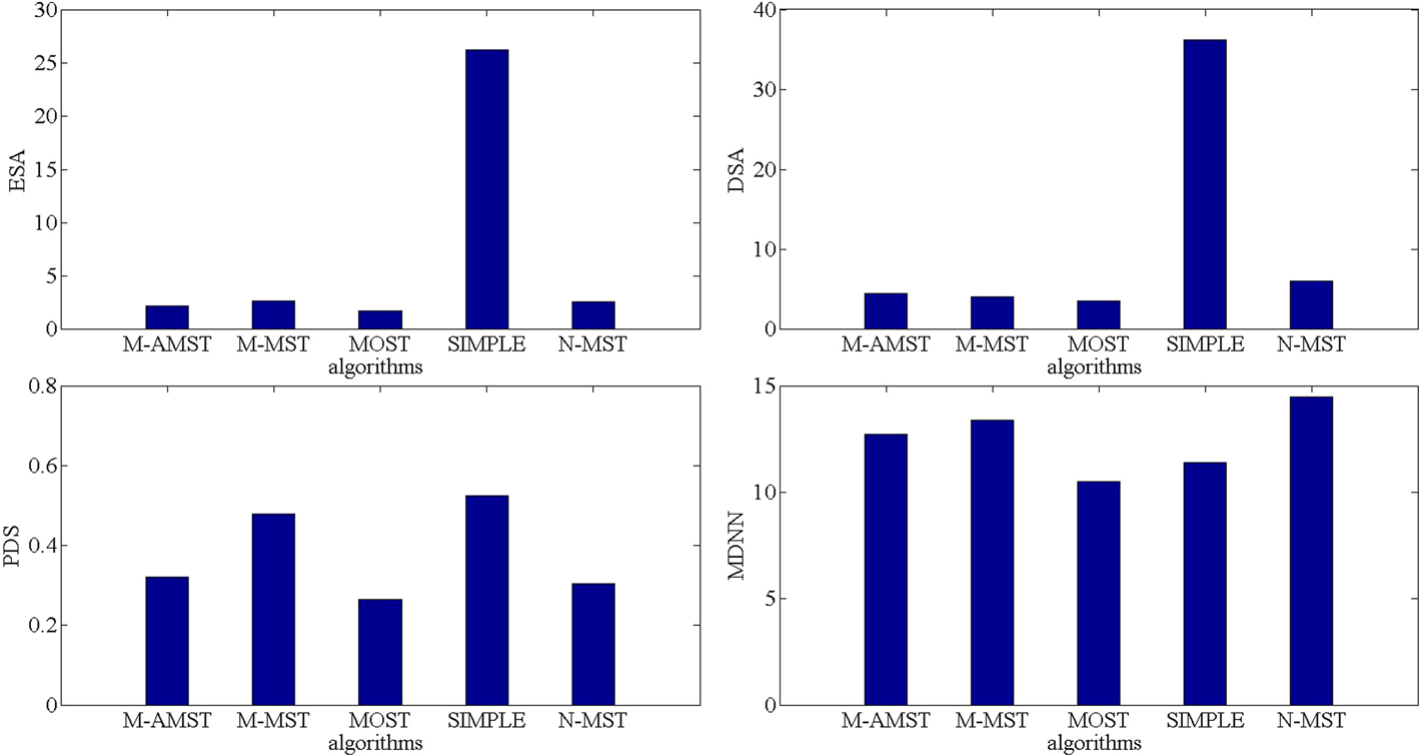
The average of the four difference scores of the five algorithms compared with APP2 reconstructions

**Fig. 6:**
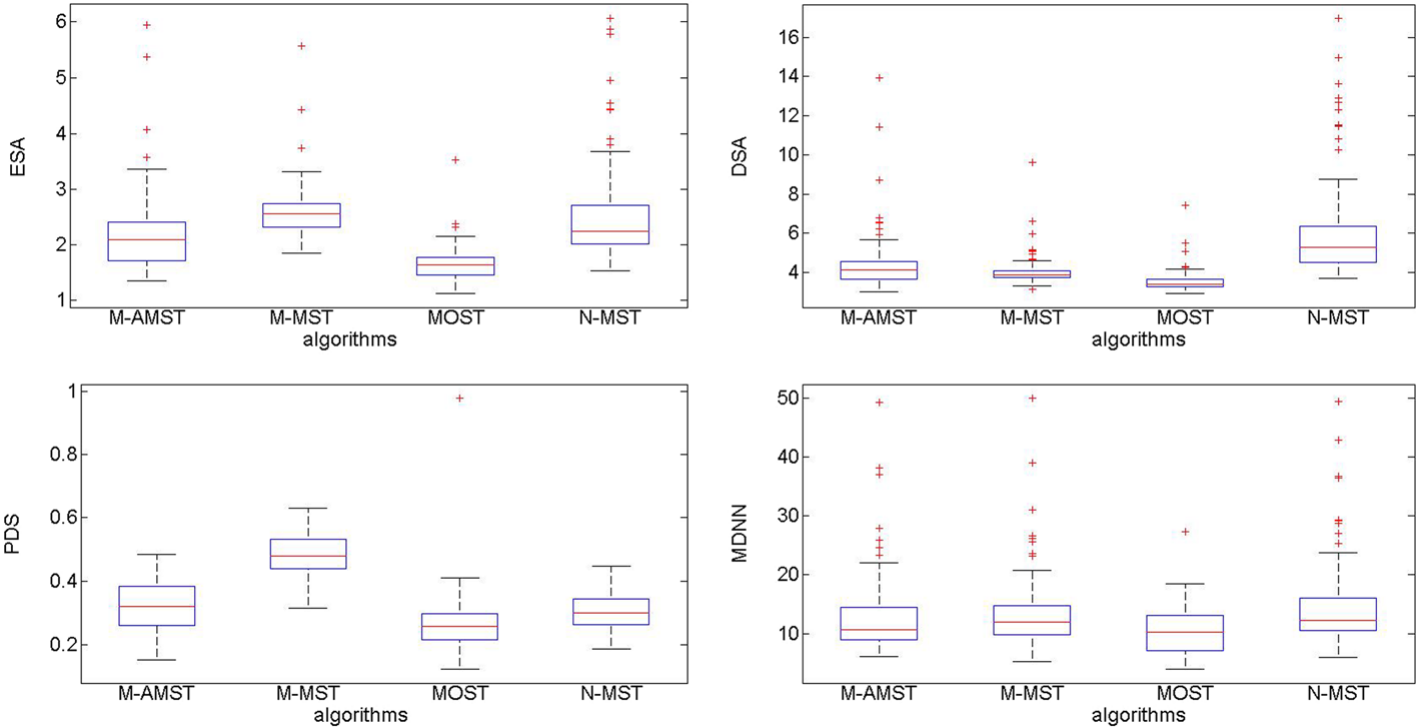
The box plots of the four difference scores of the neuron reconstructions obtained by the four neuron tracing algorithms

The histogram and boxplot are adopted to compare the performance of the five algorithms. Figure 5 shows the average of the four difference scores of the five algorithms compared with APP2 reconstructions. As we can see, MOST achieves the best reconstructions and the M-AMST gets the relative good results. Comparing with M-MST, M-AMST gets the lower average of difference score in ESA, PDS and MDNN. Although the average of DSA of M-AMST is lower than M-MST, the two values are very close. Moreover, comparing with N-MST, the average of three difference scores of M-AMST includes the ESA, DSA and MDNN are better. It indicates that utilizing the adapted MST proposed in this paper can achieve better neuron reconstructions. For both of the M-AMST and M-MST, the average of the percentage of different structure are lower than N-MST. This probably due to the node pruning method leads to the effect of over-deletion which means the method does not keep the sufficient nodes to delineate the whole topology. In order to illustrate the distribution of the four difference scores of the five competing algorithms, Fig. 6 shows the box plots of the four difference scores of the neuron reconstructions obtained by the algorithms. Due to the average of four difference scores of SIMPLE is far beyond other four competing algorithms, list each difference score of the four competing algorithm with the SIMPLE’s together will decrease the observability of the figure. Therefore, Fig. 6 only shows the difference score of the four algorithms. From the distribution of the four difference scores show in the Fig. 6, we can get that compare with the M-MST and N-MST, the ESA and MDNN score of M-AMST is better, the number of the corresponding outlier is also less, which means the distance between the reconstruction of the M-AMST and APP2 is smaller than the distance between the reconstruction of the APP2 and the M-MST or N-MST, this fully proves the effective of the adapted MST method. However, from the DSA and PDS aspects, the M-AMST does not get the best scores. Moreover, the four difference scores of the MOST are better than the M-AMST, which might be attributable to the MOST is better at reconstructing the confocal neuron images of the Drosophila. In order to further explain the effect of the M-AMST, we tested M-AMST on serval neuron datasets and compared the M-AMST reconstructions with the gold standard reconstructions.

#### D. Tracing different neuron images and comparing with the gold standard reconstruction

We test the 6 algorithms (M-AMST, M-MST, MOST, SIMPLE, N-MST and APP2) on the 7 neuron datasets respectively. The 7 datasets were one part of the data released in the first bench-testing of the BigNeuron which was started in the summer of 2015. The neuron images released in the first bench-testing phase are all with the gold standard reconstruction and aimed at fine-tuning the algorithms or training the classifiers of the BigNeuron contributor. Table 2 summarizes the basic information of the 7 neuron datasets and the reconstruction result of the 6 algorithms. (1)-(7) represents the dataset of checked6 frog scripts, checked6 human culturedcell Cambridge in vitro confocal GFP, checked6 janelia flylight part2, checked6 zebrafish horizontal cells UW, checked7 janelia flylight part1, checked7 taiwan flycirciut and checked7 utokyo fly respectively. Correspondingly, the number of the neuron images included in the each dataset is orderly indicated by N1-N7. N8 means the number of the neuron reconstructions successfully generated by each algorithm. The four difference scores (ESA, DSA,PDS and MDNN) are denoted by a-d in order, their values are obtained by comparing the SWC file of the reconstruction algorithm with the gold standard SWC file. According to the number of the neuron images included in each dataset, three representations are adopted to illustrate the result of the four difference scores: (1) each difference score is illustrated by a float if the dataset includes one image; (2) each difference score is represented by the average and the standard variance if the dataset includes more than or equal to two images; (3) each difference score is denoted by “-” if the reconstruction algorithm fails to reconstruct the neuron or generate the SWC file. The four difference scores marked with the red bold font indicates that the related algorithm achieves the best score result compare with other algorithms. The four difference score marked with the black bold font means that the related algorithm gets the second-best score result. As is shown in the table, for the dataset of (4), (5) and (6), the M-AMST gets the best difference scores. For the dataset of (1) and (3), the M-AMST achieves the second-best difference scores. For the dataset of (1), the M-AMST is close to APP2 in the ESA, and the M-AMST is superior to APP2 in PDA. For the dataset of (2), the MOST obtains the best difference scores. However, 6 algorithms are all failed to get the good reconstructions, which means there is no significance to compare the reconstruction effect of the algorithms for this kind of dataset. For the dataset of (3), N-MST gets the best difference scores, after observing the raw image, we find the neurons in the image are all surround many white spots with low intensity and high density. As mentioned above, the M-AMST adopts a rigorous de-noise method to identify the foreground voxel in the binarization step, which causes many noisy voxels are deemed to be the foreground and makes M-AMST is inferior to APP2 in DSA and PDS. To sum up, using the gold standard SWC file as the reference and making a comparison between M-AMST and the other 5 algorithms, M-AMST is able to reconstruct variety of neuron datasets successfully and achieve the good difference scores.

**Table 2:**
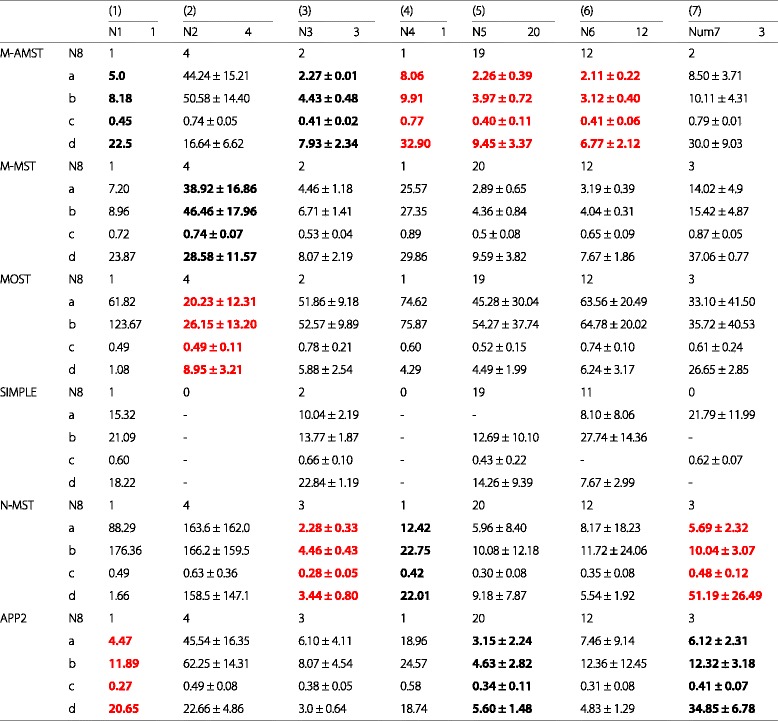
The basic information of the 7 neuron datasets and the reconstruction result of the 6 algorithms

The symbol “-” means the reconstruction algorithm fails to reconstruct the neuron or generate the SWC file. The four difference scores marked with the red bold font indicates that the related algorithm achieves the best score result compare with other algorithms. The four difference score marked with the black bold font means that the related algorithm gets the second-best score result

## Discussion

In summary, the main steps of the M-AMST followed the sequence of neuron reconstruction operations: binarization, skeletonization, rectification and graph representation. In the skeletonization step, M-AMST adopts mean shift to extract nodes distributed around the centroid of the local neuron segments. The reason for using mean shift can be concluded as the two aspects. On one hand, mean shift is an automatic clustering algorithm which can move and cluster each foreground voxel to the local convergence region. The voxels located at the convergence region cannot shift for one step by mean shift. By observing their position in the neuron image, the most classification voxels are located around the neuron skeleton, in which the neuron portions with high intensity and voxel density located. On the other hand, other voxels which shift toward and finally locate at the regions after several iterations can be marked as the same classification as the corresponding classification voxels. That kind of voxels can be regarded as the subordinate voxels to the classification voxels. Based on the subordinate voxels belong to the different classification voxel, the local structure of the neuron can be delineated. Therefore, with mean shift, not only the information of voxel density distribution of neuron image can be captured correctly, but also got the sufficient voxels belong to several modes to delineate the whole neuron topological structure. Moreover, we develop a pathway extraction method using a rotating sphere model based on coordinate transformation to improve the weight calculation approach in MST. It is worth noting that the pathway contains a node list since selecting the voxel which intensity is greater than 0 as the new sphere center in every rotating step. Every node list represents the shape information between the corresponding pair of nodes. For a pathway between the pair of nodes, we calculate the length of it by sequentially accumulating the Euclidean distance between the parent node and child node in the list. And then, take the accumulating Euclidean distance as the weight of edge. Notable, the weight will be set to a numerical value which is far greater than the real spatial distance if the sphere rotating model fails to extract the pathway. In the experimental stage, two experiments are designed illustrate the effectiveness of the adapted MST method and the adoptability of M-AMST in reconstructing variety of neuron datasets. The result of experiment 1 shows that M-AMST gets lower difference scores than M-MST in ESA, PDS and MDNN. Meanwhile, M-AMST is better than N-MST in ESA and MDNN. It indicates that utilizing the adapted minimum spanning tree algorithm which took the shape information of neuron into account can achieve better neuron reconstructions. In order to testify the adoptability of M-AMST, 7 neuron datasets released in the first bench-testing stage of the BigNeuron are chose to be reconstructed by 6 algorithms in the experiment 2. The neuron reconstruction results generated by each algorithm are compared with the corresponding gold standard SWC file. The comparison result shows that M-AMST is able to achieve the best difference score in 3 datasets and get the second-best difference score in the other 2 datasets. This indicates that the M-AMST can reconstruct variety of neuron datasets successfully and achieve good difference scores. However, there are still several limitations in M-AMST which are listed as follows.

1. Several neuron segments with low intensity are not reconstructed successfully. The reason can be concluded into two points. On one hand, the foreground identification method used in the binarization step might ignore the nodes with low intensity. On the other hand, the voxels with low intensity located around the neuron skeleton will be moved in several iterations and cannot be identified as the classification marks.
2. After analyzing the reconstructions generated by M-AMST thoroughly, we find that M-AMST did not thoroughly solve the problem of connecting the different segments by crossing the gap. The main reason for this is the pathway extraction method failed when there is a gap between the pair of nodes. In this case, although a numerical value which is far greater than the real spatial distance is assigned to the weight of edge between them, MST might still select this pathway if there is no smaller weight can be chose.
3. The node pruning method probably degrades the reconstruction accuracy of M-AMST. For each pair of nodes, we define one of the pair of nodes should be deleted if the difference between their Euclidean distance D and the sum of their radiuses is less than a threshold. A higher threshold could cause the method cannot keep the sufficient nodes to reconstruct the whole topology. Conversely, a lower threshold could cause the reconstruction with redundant neuron segments due to the method keeps the excessive nodes.

In response to limitations mentioned above, the implementation details of M-AMST algorithm will be further refined. In the near future, we will keep working on developing the neuron image de-noising and tracing algorithms based on the machine learning method so as to continue making contributions to the BigNeuron project.

## Conclusions

We develop a pathway extraction method using a rotating sphere model based on coordinate transformation to improve the weight calculation approach in MST. The corresponding experiment shows that utilizing the adapted minimum spanning tree algorithm which took the shape information of neuron into account can achieve better neuron reconstructions. Moreover, the adoptability of M-AMST in reconstructing variety of neuron images is also testified. The result indicates that comparing with the gold standard reconstruction, the neuron reconstruction generated by the M-AMST is able to achieve good difference scores in variety of neuron datasets.

## Abbreviations

APP2: Improved all-path pruning method
M-AMST: Mean shift and adapted minimum spanning tree
M-MST: Mean shift and minimum spanning tree
MOST: Micro-Optical Sectioning Tomography ray-shooting
N-MST: Neuron tracing minimum spanning tree
p.d.f: probability density function

## References

[1] Meijering E (2010). Neuron tracing in perspective. Cytometry A.

[2] Zhong N, Yau SS, Ma J, Shimojo S, Just M, Hu B, Wang G, Oiwa K, Anzai Y (2015). Brain Informatics-Based Big Data and the Wisdom Web of Things. IEEE Intell Syst.

[3] Peng H (2008). Bioimage informatics: a new area of engineering biology. Bioinformatics.

[4] Ascoli GA (2008). Neuroinformatics Grand Challenges. Neuroinformatics.

[5] Peng H, Meijering E, Ascoli GA (2015). From DIADEM to BigNeuron. Neuroinformatics.

[6] Peng H, Hawrylycz M, Roskams J, Hill S, Spruston N, Meijering E, Ascoli Giorgio A (2015). BigNeuron: large-scale 3d neuron reconstruction from optical microscopy images. Neuron.

[7] Pal SK. Fuzzy sets in image processing and recognition. In: Fuzzy Systems, 1992, IEEE International Conference on: 8-12 Mar 1992 1992. 119-126.

[8] Malladi R, Sethian JA. Level set and fast marching methods in image processing and computer vision. In: Image Processing, 1996 Proceedings, International Conference on: 16-19 Sep 1996 1996. 489-492 vol.481.

[9] Xiao H, Peng H (2013). APP2: automatic tracing of 3D neuron morphology based on hierarchical pruning of a gray-weighted image distance-tree. Bioinformatics.

[10] Peng H, Long F, Myers G (2011). Automatic 3D neuron tracing using all-path pruning. Bioinformatics.

[11] Wang Y, Narayanaswamy A, Tsai C-L, Roysam B (2011). A broadly applicable 3-D neuron tracing method based on open-curve snake. Neuroinformatics.

[12] Cai H, Xu X, Lu J, Lichtman JW, Yung SP, Wong ST (2006). Repulsive force based snake model to segment and track neuronal axons in 3D microscopy image stacks. Neuroimage.

[13] Peng H, Ruan Z, Atasoy D, Sternson S (2010). Automatic reconstruction of 3D neuron structures using a graph-augmented deformable model. Bioinformatics.

[14] Cai H, Xu X, Lu J, Lichtman J, Yung SP, Wong ST (2008). Using nonlinear diffusion and mean shift to detect and connect cross-sections of axons in 3D optical microscopy images. Med Image Anal.

[15] Oliver A, Munoz X, Batlle J, Pacheco L, Freixenet J. Improving Clustering Algorithms for Image Segmentation using Contour and Region Information. In: 2006 IEEE International Conference on Automation, Quality and Testing, Robotics: 25-28 May 2006 2006. 315-320.

[16] Wu J, He Y, Yang Z, Guo C, Luo Q, Zhou W, Chen S, Li A, Xiong B, Jiang T (2014). 3D BrainCV: Simultaneous visualization and analysis of cells and capillaries in a whole mouse brain with one-micron voxel resolution. NeuroImage.

[17] Yang J, Gonzalez-Bellido PT, Peng H (2013). A distance-field based automatic neuron tracing method. BMC Bioinformatics.

[18] Comaniciu D, Meer P (2002). Mean shift: a robust approach toward feature space analysis. IEEE Trans Pattern Anal Mach Intell.

[19] Peng H, Ruan Z, Long F, Simpson JH, Myers EW (2010). V3D enables real-time 3D visualization and quantitative analysis of large-scale biological image data sets. Nat Biotech.

